# Prophylactic cranial irradiation reduces the incidence of brain metastasis in a mouse model of metastatic, HER2-positive breast cancer

**DOI:** 10.18632/genesandcancer.212

**Published:** 2021-03-13

**Authors:** Daniel L. Smith, Bisrat G. Debeb, Parmeswaran Diagaradjane, Richard Larson, Swaminathan Kumar, Jing Ning, Lara Lacerda, Li Li, Wendy A. Woodward

**Affiliations:** ^1^Department of Radiation Oncology, University of Texas MD Anderson Cancer Center, Houston, TX, USA; ^2^Department of Biostatistics, University of Texas MD Anderson Cancer Center, Houston, TX, USA; ^3^Morgan Welch Inflammatory Breast Cancer Research Program and Clinic, University of Texas MD Anderson Cancer Center, Houston, TX, USA; ^4^The University of Texas MD Anderson Cancer Center, UTHealth Graduate School of Biomedical Sciences, Houston, TX, USA

**Keywords:** prophylactic cranial irradiation, brain metastasis, breast cancer

## Abstract

Prophylactic cranial irradiation (PCI) can reduce the incidence of brain metastasis and improve overall survival in some patients with acute lymphoblastic leukemia or small-cell lung cancer. We examined the potential effects of PCI in a mouse model of breast cancer brain metastasis. The HER2+ inflammatory breast cancer cell line MDA-IBC3 was labeled with green fluorescent protein and injected via tail-vein into female SCID/Beige mice. Mice were then given 0 Gy or 4 Gy of whole-brain irradiation 2 days before tumor-cell injection or 5 days, 3 weeks, or 6 weeks after tumor-cell injection. Mice were sacrificed 4-weeks or 8-weeks after injection and brain tissues were examined for metastasis by fluorescent stereomicroscopy. In the unirradiated control group, brain metastases were present in 77% of mice at 4 weeks and in 90% of mice at 8 weeks; by comparison, rates for the group given PCI at 5 days after tumor-cell injection were 20% at 4 weeks (*p*=0.01) and 30% at 8 weeks (*p*=0.02). The PCI group also had fewer brain metastases per mouse at 4 weeks (*p*=0.03) and 8 weeks (*p*=0.006) versus the unirradiated control as well as a lower metastatic burden (*p*=0.01). Irradiation given either before tumor-cell injection or 3-6 weeks afterward had no significant effect on brain metastases compared to the unirradiated control. These results underscore the importance of timing for irradiating subclinical disease. Clinical whole brain strategies to target subclinical brain disease as safely as possible may warrant further study.

## INTRODUCTION

Breast cancer brain metastasis is a significant clinical problem. Despite improvements in multimodal therapy, only 20-30% of patients with breast cancer will survive for longer than one year after the diagnosis of brain metastasis. Moreover, as the population ages and methods for extracranial disease control continue to improve, the number of patients with breast cancer who will develop and die from brain metastases continues to rise [1, 2]. 

One strategy that could improve outcomes for such patients is prophylactic cranial irradiation (PCI), defined as whole-brain irradiation given in an effort to eradicate micrometastatic disease in the brain before it grows into overt disease. PCI has been used for decades for patients with small cell lung cancer and children with acute lymphoblastic leukemia; in both cases, PCI significantly reduces the incidence of brain metastasis and improves overall survival [3-6]. In patients with non-small cell lung cancer, PCI significantly reduced the incidence of brain metastasis, but to date has not led to improved overall survival, possibly because of underpowered studies, inadequate imaging for patient selection, or systemic failures [7, 8]. Interest is increasing in using PCI to prevent brain metastasis in patients with breast cancer, but to date only two small studies have been conducted [9-11]. The principal drawback of PCI is the potential for toxicity, including fatigue and hair loss during treatment and neurocognitive decline among patients with prolonged survival [12]. However, new strategies to reduce toxicity including use of memantine and hippocampal sparing intensity modulated radiation techniques are becoming more widely used in clinical practice for patients in whom whole brain radiation remains first line therapy [13, 14]. 

**Figure 1:**
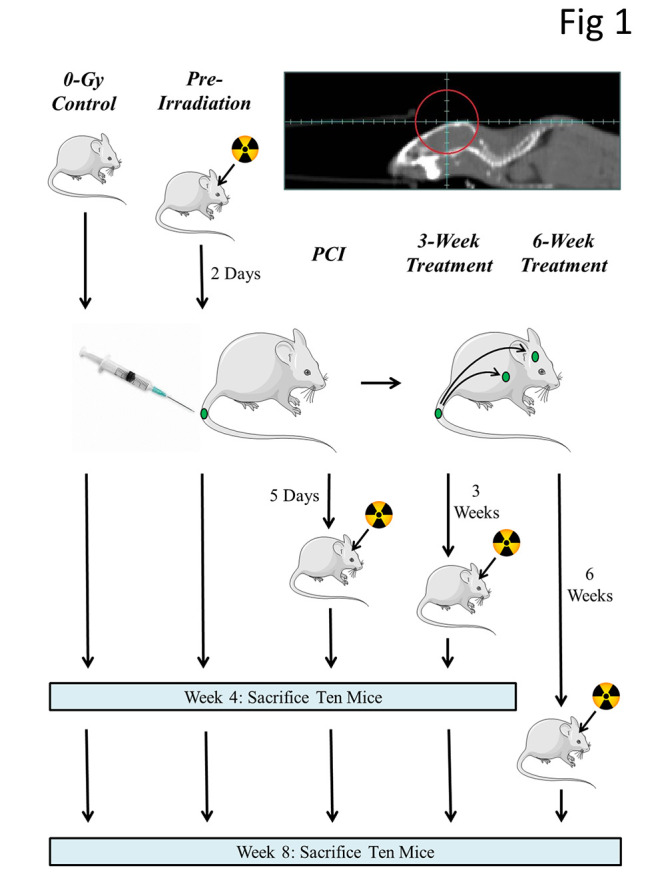
Experimental design. SCID/Beige mice were injected, via tail vein, with 5 × 105 GFP-labeled MDA-IBC3 cells and irradiated with a single 4-Gy fraction (opposing lateral fields as indicated by the red circle) at the indicated times; mice were sacrificed at 4 weeks or at 8 weeks after tumor-cell injection and their brain was examined for the presence of metastases.

**Table 1:** Table 1: Incidence of Brain Metastasis at the Four-Week Endpoint

**Dose**	**Time of Irradiation**	**Incidence**	**%**	***p*-value vs. PCI**
0 Gy	-	10/13	77%	0.01
4 Gy	2 days pre-injection	10/10	100%	0.0007
4 Gy	5 days post-injection [PCI*]	2/10	20%	-
4 Gy	3 weeks post-injection	7/10	70%	0.07

*Prophylactic cranial irradiation

The principal factor in determining whether the potential benefit of PCI (increased survival) would outweigh the potential risks (morbidity) would be the risk of developing brain metastases. Among all breast cancer patients, that risk is 5-10%, but the risk increases to 15% among patients with extracranial metastases (stage IV disease) [15, 16]. The risk increases further for breast cancer of specific receptor subtypes: patients with human epidermal growth factor receptor 2-enriched (HER2+) or triple-negative stage IV breast cancer are at a 25-45% risk of developing brain metastasis [17, 18]. Clinical factors, applied in a nomogram, and the identification of biomarkers could also help to identify which such patients are at the highest risk of developing brain metastases [19]. In addition, it is noted that if toxicity could be reduced with techniques above or even lowered dose, further consideration could be given to selection of patients for whole brain after stereotactic radiosurgery where the risk of further subclinical disease is expected.

Because patients with stage IV HER2+ or triple-negative breast cancer are at particularly high risk of developing brain metastasis and because the prognosis of patients who develop brain metastases is very poor, we investigated whether low dose PCI could reduce the incidence of brain metastasis in a mouse model of metastatic breast cancer, in which tail-vein injection of HER2+ breast cancer cells led to a high rate of brain metastasis [20]. Advances in small-animal radiation research [21] have allowed us to reproducibly administer whole-brain irradiation to dozens of mice without significant morbidity. Here, we show that a dose of radiation delivered 5 days after injection of tumor cells reduces both the incidence of brain metastasis and metastatic burden, but delayed treatment has no observable effect. 

## RESULTS

**Figure 2:**
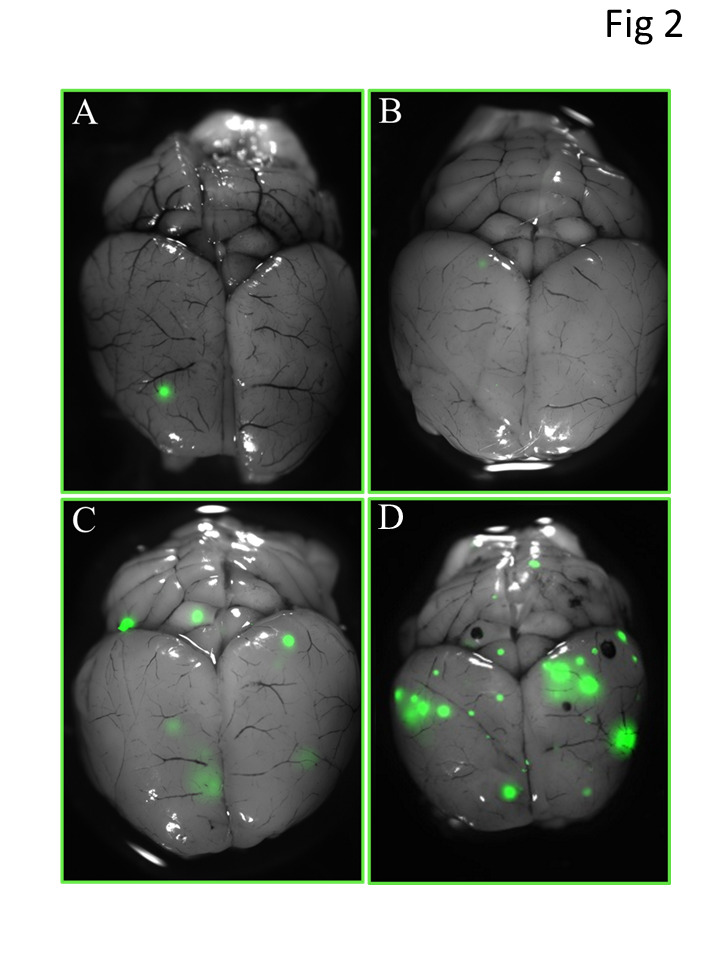
Images of brain metastases from PCI and treatment groups at 8 weeks after injection of GFP-labeled tumor cells. Brain images were obtained with a fluorescent stereomicroscope. Panels A and B show representative images with (A) and without (B) brain metastases after receiving 4 Gy of whole-brain irradiation 5 days after tumor-cell injection; panel C, image from a mouse that received 4 Gy whole-brain irradiation at 3 weeks after tumor-cell injection; and panel D, image from a mouse that received 4 Gy of whole-brain irradiation 6 weeks after tumor-cell injection. Metastatic foci were the smallest in the mice irradiated 5 days after tumor-cell injection.

To assess the effects of PCI in a mouse model of breast cancer brain metastasis, we subjected mice to whole-brain irradiation at different times before or after having been injected with 5 × 10^5^ GFP-labeled MDA-IBC3 cells and then sacrificing the mice at either 4 weeks (n=43) or 8 weeks (n=45) later. These endpoints were chosen to examine if any effect of PCI observed at 4 weeks was transitory (e.g., reflecting a delay in tumor growth) or persistent. Excised brain tissue from each mouse was evaluated for the presence of GFP-labeled metastases; representative brain images from mice treated at various times after tumor-cell injection and sacrificed at 8 weeks are depicted in Figure 2. (Images of brain metastases at 4 weeks are shown in Supplementary Figure S1; and images of brain sections stained with hematoxylin and eosin are shown in Supplementary Figure S2.) 

The rates of brain metastasis among the five treatment groups at 4 weeks and at 8 weeks after tumor-cell injection are shown in Table 1. The group given 4 Gy of whole-brain irradiation at 5 days after tumor-cell injection had the lowest incidence of brain metastasis at both endpoints, supporting our hypothesis that PCI (defined as irradiation given at 5 days after tumor-cell injection; based on the *in vitro* doubling time of MDA-IBC3 cells, we would not expect to observe brain metastases five days after cell injection) would reduce the incidence of brain metastasis. These differences held when the data from both the 4-week and 8-week endpoints were combined (Supplementary Table S1). 

**Table 2:** Table 2: Incidence of Brain Metastasis at the Eight-Week Endpoint

**Dose**	**Time of Irradiation**	**Incidence**	**%**	***p*-value vs. PCI**
0 Gy	-	9/10	90%	0.02
	2 days pre-injection	10/10	100%	0.003
4 Gy	5 days post-injection [PCI*]	3/10	30%	-
	3 weeks post-injection	7/7	100%	0.009
	6 weeks post-injection	7/8	88%	0.02

**Prophylactic cranial irradiation

**Figure 3:**
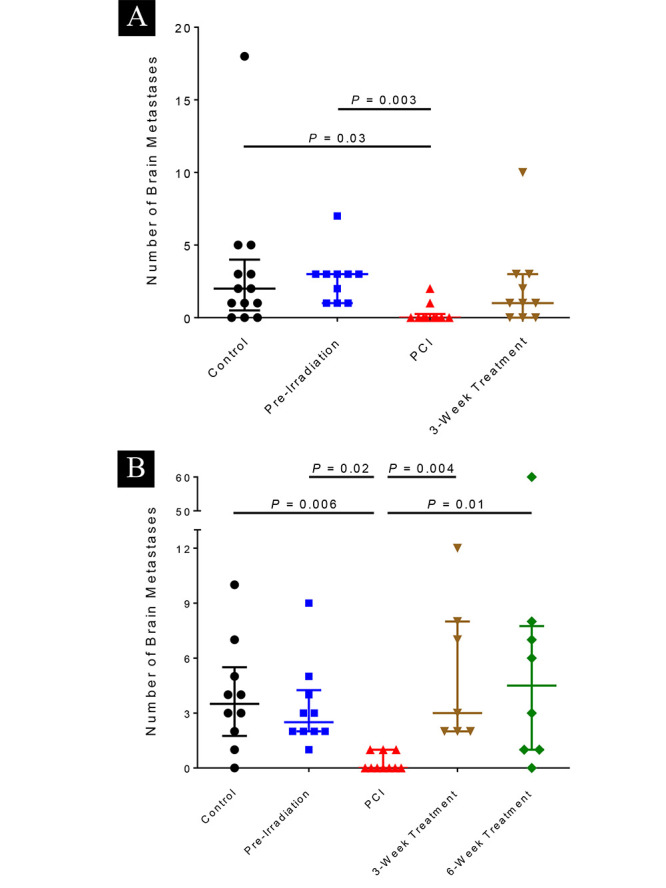
Numbers of brain metastases per mouse at 4 weeks (top) or at 8 weeks (bottom) after tumor-cell injection. Brain metastases were identified with a fluorescent stereomicroscope and the number of brain metastatic foci per mouse was counted. At 4 weeks, the group given whole-brain irradiation at 5 days after tumor-cell injection (prophylactic cranial irradiation [PCI]) had significantly fewer brain metastases per mouse than did the control group and the group given irradiation 2 days before tumor-cell injection (pre-irradiation). At 8 weeks, the group that was given whole-brain irradiation 5 days after tumor-cell injection (PCI group) had significantly fewer brain metastases per mouse than any of the other groups. Horizontal bars represent medians and lower/upper quartiles.

**Figure 4:**
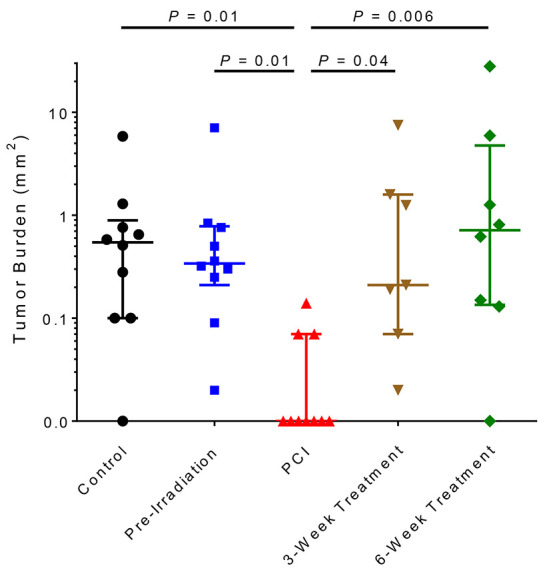
Brain metastasis burden at 8 weeks after tumor-cell injection. Eight weeks after tumor-cell injection, tumor burden per mouse was calculated with Nikon NIS-Elements software. The group that was given whole-brain irradiation at 5 days after tumor-cell injection (PCI group) had the lowest tumor burden of any of the groups. Horizontal bars represent median and lower/upper quartiles.

The number of brain metastases per mouse was also significantly reduced in the mice irradiated at 5 days after tumor-cell injection at both the 4-week and 8-week endpoints (Figure 3). At the 4-week endpoint, those mice (PCI group) had significantly fewer metastases than the control group (*p*=0.03) or the group irradiated before tumor-cell injection (pre-irradiated group, *p*=0.003). At the 8-week endpoint, mice in the PCI group had significantly fewer metastases than any other groups. The PCI group also had the lowest tumor burden of any of the groups at 8 weeks (Figure 4). No differences were observed in the body weight of mice among the different treatment groups (Supplementary Figure S3), nor in the incidence of lung metastases examined at the four-week time point (data not shown). 

## DISCUSSION

Here, we demonstrated that low-dose prophylactic cranial irradiation (PCI) significantly reduced the incidence and burden of brain metastases in a mouse model of HER2+ breast cancer. Moreover, the incidence did not increase from 4 weeks to 8 weeks after tumor-cell injection, suggesting that this treatment produced a persistent, long-lasting decrease in metastasis rather than merely delaying their onset. The same radiation dose (4 Gy) had no observable effect when the whole-brain irradiation was given either before or 3-6 weeks after tumor-cell injection.

PCI not only reduced the incidence of brain metastasis but also suppressed both the number of metastases and the overall metastatic burden. Two mice in the PCI group developed brain metastases at 4 weeks and three others in the PCI group had brain metastases at eight weeks; among those five mice (out of 20 total), there were collectively six brain metastases (Table 1). By contrast, 19 mice in the control group (out of 23 total) had collectively 80 brain metastases. This >90% reduction in number of metastases in the PCI group is inconsistent with our unpublished *in vitro* results, where the clonogenic survival of MDA-IBC3 cells ranges from 25% to 80% after 4 Gy depending on cell culture conditions. These findings suggest that radiation may affect a relatively late step in the metastatic process, such as colonization.

Our finding of a reduced metastatic burden at 8 weeks in the PCI group (that is, mice given radiation at 5 days after tumor-cell injection) in part reflects the presence of fewer brain metastases; however, the three metastases present in the PCI group at 8 weeks were all relatively small. This was an unexpected finding, as cells that retain their clonogenic potential after early irradiation would still have the full 8 weeks to grow. Indeed, the mice irradiated 3 and 6 weeks after tumor-cell injection were included as another control, in that one would expect brain metastases to already be present by the time those mice were irradiated. No differences were noted between the unirradiated control and these two delayed-irradiation groups. 

Finally, we attempted to control for radiation effects on the local microenvironment by including an experimental group that was irradiated 2 days before cell injection. Barcellos-Hoff and colleagues [23] found that non-transformed mammary epithelial cells preferentially formed tumors in cleared mammary fat pads that had been pre-irradiated with 4 Gy. In our study, 100% of mice developed brain metastasis at both endpoints; however, this was not significantly different than the unirradiated controls, and little difference was found in comparing the number of brain metastases per mouse and the metastatic burden. These findings suggest that in the PCI group, the effect of radiation on the microenvironment was not a major contributor to the lower incidence and burden of brain metastasis. 

This study had several limitations. We used a tail-vein injection mouse model rather than a spontaneous model, meaning that a single bolus of breast cancer cells entered the circulation rather than being shed from the primary or metastatic sites over time; moreover, only single cells entered the circulation, which may not recapitulate the clinical situation [24-26]. Further these tail-vein injected cells must escape from the pulmonary circulation which differs from more commonly used cardiac injection models, but may be biologically relevant. Next, we used a single 4-Gy dose, but patients who receive whole-brain irradiation usually receive several fractions. Nevertheless there was no toxicity observed among irradiated animals, and this dose is similar to a typical daily fraction of radiation (30 Gy in ten fractions or 20 Gy in five in patients with low performance status). 

Despite these limitations, this study highlights the importance of timing in the treatment of subclinical disease. The additional 16 days of growth between the PCI and the first delayed treatment group led to substantial differences in the incidence of brain metastasis, number of metastases, and metastatic burden (Table 2). This finding is analogous to results from a meta-analysis reported by Suwinski et al. [27] regarding PCI dose-response in small cell lung cancer. In that analysis, “early” PCI, in which PCI was begun <60 days after treatment of the primary tumor was begun, was compared with “late” PCI. Although brain relapse rates were reduced even at low doses in the “early” group, a dose threshold of 20 Gy was evident in the “late” PCI (>60 days) group, consistent with the growth of untreated subclinical disease during the interval between treatments. 

The use of whole-brain irradiation as a prophylactic or to treat asymptomatic brain metastases in patients with breast cancer has been limited. In one study [10], 10 patients with stage IIIB/IV breast cancer in continued remission received PCI at a dose of 36 Gy in 20 fractions. Although only 2 out of 10 patients developed brain metastases in the PCI, 3 patients with prolonged survival showed serious neurocognitive declines. A separate study [28] compared the efficacy of whole-brain radiation therapy – 30 Gy in 10 fractions – between patients with symptomatic brain metastases and patients with asymptomatic brain metastases. Only 16% of the patients in the asymptomatic group died of progressive brain disease (vs. 48% in the symptomatic group) but no difference was found in overall survival, likely from failure to control extracranial disease. Finally, a more recent report described a phase III trial to study PCI for patients with locally advanced or metastatic HER2+ breast cancer that had relapsed after trastuzumab treatment [9]. The recruitment of 51 patients fell far short of the 390-patient target, and thus the apparent reduction in the incidence of brain metastasis at 2 years in the PCI group (21% after 30 Gy in 10 fractions vs. 32% in the non-PCI group) was not statistically significant.

Given the evident importance of the timing of irradiation, it may be beneficial to refer patients at high risk of developing brain metastases for scans as part of their continued management. A follow-up question would be whether patients with negative brain scans should receive PCI. The design of potential PCI clinical trials for these patients would involve several factors, most notably patient selection in light of the risk of toxicity from whole-brain irradiation. Patients with stage IV HER2+ triple-negative breast cancer are the most likely to develop brain metastases, and the nomogram developed by Ibrahim and colleagues [19] may be a good starting point to select individual patients for trials. If PCI were restricted to patients with controlled extracranial disease, then re-seeding of the brain from extracranial disease would not be an immediate concern, and improvements in intracranial control could lead to improvements in survival. We speculate that if a low dose were effective in the PCI setting, repeated low-dose PCI after new re-seeding suspected at the time of progression could be both feasible and safe. Emerging strategies to reduce the toxicity of whole brain radiation such as concurrent memantine and hippocampal brain sparing techniques further the cause to explore the role of PCI in these patients [13, 14]. 

Our findings also have implications for whole-brain radiation therapy given after stereotactic radiosurgery (SRS). Recent findings suggest that patients who receive such treatment after SRS had better intracranial control but no overall survival benefit and significant cognitive morbidity [29, 30]. Our experimental data highlight the need to understand the timing of extracranial tumor shedding and the potential for dose de-escalation to mitigate the morbidity and confounders with whole brain radiation therapy (WBRT). Although we have not experimentally addressed whether the PCI is affecting colonization or growth in this study, the companion modeling work suggests the effect is through cell kill, not altered colonization [31]. 

## MATERIALS AND METHODS

### Cell culture 

Tumor cells used for these experiments were the HER2+ inflammatory breast cancer cell line MDA-IBC3, generated as described elsewhere [22]. Cells were cultured in Ham’s F-12 medium supplemented with 10% fetal bovine serum, 1 µg/mL hydrocortisone, 5 µg/mL insulin, and 1% antibiotic-antimycotic, maintained in a humidified, 5% CO2 environment at 37°C, and passaged approximately every 4 days. This cell line has been verified as negative for mycoplasma contamination by the Lonza MycoAlert Mycoplasma Detection kit. These cells had previously been transfected with a plasmid (Systems Biosciences) that encodes for green fluorescent protein (GFP), which was then transduced via lentivirus as described previously [22]. 

### Tail-vein injection 

Three- to five-week-old female immunocompromised SCID/Beige mice (Harlan, USA) were housed and used in accordance with the institutional guidelines of MD Anderson Cancer Center under a protocol approved by the Institutional Animal Care and Use Committee (ACUF 00001142-RN00). 

MDA-IBC3 cells labeled with GFP were cultured *in vitro* as described above until 60-70% confluence, after which they were treated with trypsin, counted, and intravenously injected into the mice via the tail vein (5 × 10^5^ cells in 200 μL phosphate-buffered saline per mouse). Mice were euthanized (using CO_2_) and subjected to necropsy at either 4 weeks or at 8 weeks after injection of the tumor cells. Brain metastatic colonization was evaluated by fluorescent stereomicroscopy. Mice injected with cancer cells but either died immediately or days after injection or were found dead before the 4-week or 8-week endpoint were excluded from the final analysis. 

### Whole-brain irradiation 

Five groups of mice received whole-brain irradiation in a small-animal irradiator at different times with respect to tumor-cell injection (Figure 1). The control (unirradiated) group (n=23) received 0 Gy (13 mice were sacrificed at 4 weeks after tumor-cell injection and 10 at 8 weeks); the second group (n=20) was irradiated 2 days before tumor-cell injection (10 sacrificed at each time point); in the third group, mice (n=20) were irradiated 5 days after tumor-cell injection (10 sacrificed at each time point); in the fourth group, mice (n=17) were irradiated 3 weeks after tumor-cell injection (10 sacrificed at 4 weeks and 7 sacrificed at 8 weeks); and in the fifth group, mice (n=8) were irradiated 6 weeks after tumor-cell injection (all 8 were sacrificed at 8 weeks). 

For treatment planning and irradiation, mice were anesthetized with isoflurane and placed in the imaging and treatment stage of an X-RAD 225Cx small-animal irradiator (PRECISION X-RAY, North Branford, CT, USA); cone-beam computed tomography images were obtained at 40 kVp and 2.50 mA and used to manually set the isocenter for each mouse. All mice were irradiated according to the same treatment plan, which was developed with PilotXRAD 1.10.4 software. Each mouse received a single 4-Gy fraction to the whole-brain in two 2-Gy lateral opposing fields. We selected a dose of 4 Gy due to the effects of this dose on MDA-IBC3 cells *in vitro*, where we observe an approximately 30% survival fraction (not shown). Irradiations were done at 225 kVp and 13.0 mA, with a 15-mm-diameter field size, at a dose rate of approximately 3.2 Gy per minute. Care was taken to exclude the aerodigestive tract of the mice from the treatment field. 

### Fluorescent microscopy 

Mice were euthanized at 4 or at 8 weeks after tumor-cell injection, and brain tissues were isolated and evaluated for metastatic colonization by measuring GFP levels with a Nikon AZ100 microscope (Tokyo, Japan). The primary endpoint was the presence or absence of metastases in the brain; numbers of metastases in the brain was counted as well.

Brain tumor burden was measured with the Nikon’s NIS-Elements software. The areas of each metastatic focus, visualized from either the top or bottom images of the brain, were summed to give a surrogate for the total tumor burden. Images were prepared by subtracting the autofluorescence background and overlaying the result on the corresponding photograph.

### Statistical analysis and Sample size justification 

Fisher’s exact tests were used to compare the incidence of metastatic colonization to the brain in the different groups. Ten mice in the PCI group and 13 mice in the unirradiated control group gives more than 80% power with a 2-sided α=0.05 to detect a difference in metastatic incidence rates at 4 weeks between 0.16 and 0.80 of two group, respectively. For the comparison of metastatic incidence rates at 8 weeks, 10 mice (7 mice) in the PCI group and 10 mice in the unirradiated control group can have 84% (79%) power with a 2-sided α=0.05 to detect a difference between 0.2 and 0.9 of two groups. Mice injected with cancer cells but either died immediately or days after injection or were found dead before the 4-week or 8-week endpoint were excluded from the final analysis. 

Dunn’s test was used to compare the number and burden of brain metastases between individual groups. For the comparison of numbers of brain metastases at 4 weeks, 10 mice in the PCI group and 13 mice in the unirradiated control group have 80% power with a 2-sided α=0.05 to detect a difference in means of 1.4 assuming a standard deviation of 1. For the comparison of numbers of brain metastases at 8 weeks, 10 (7 mice) mice in the PCI group and 10 mice in the unirradiated control group have 80% power with a 2-sided α=0.05 to detect a difference in means of 1.6 (1.8) assuming a standard deviation of 1. 

## CONCLUSION

In this work, we used a unique experimental system – a robust mouse model of Her2-positive breast cancer brain metastasis and a dedicated small-animal irradiator –to address the potential efficacy of PCI, which may be clinically relevant for patients with breast cancer. The median survival time for patients with breast cancer and brain metastases is well under 1 year, and patients with stage IV HER2+ or triple-negative breast cancer are at particularly high risk of developing brain metastases. Our hypothesis, that PCI would reduce the incidence of brain metastasis in a mouse model of HER2+ inflammatory breast cancer, was strongly supported. If validated and extended, these findings have the potential to inform the clinical management strategy for patients with breast cancer at high risk of developing brain metastases.

## SUPPLEMENTARY MATERIALS



## Abbreviations

PCI: prophylactic cranial irradiation
HER2+: human epidermal growth factor receptor 2-enriched
GFP: green fluorescent protein
SRS: stereotactic radiosurgery
WBRT: whole brain radiation therapy
