# Supplementary material for: Prophylactic cranial irradiation reduces the incidence of brain metastasis in a mouse model of metastatic, HER2-positive breast cancer
Source: Genes Cancer. 2021 Mar 13;12:28–38. doi: 10.18632/genesandcancer.212 (PMC8045965; doi:10.18632/genesandcancer.212)
Supplement: Supplementary file 1 [file ganc-12-28-s001.pdf]

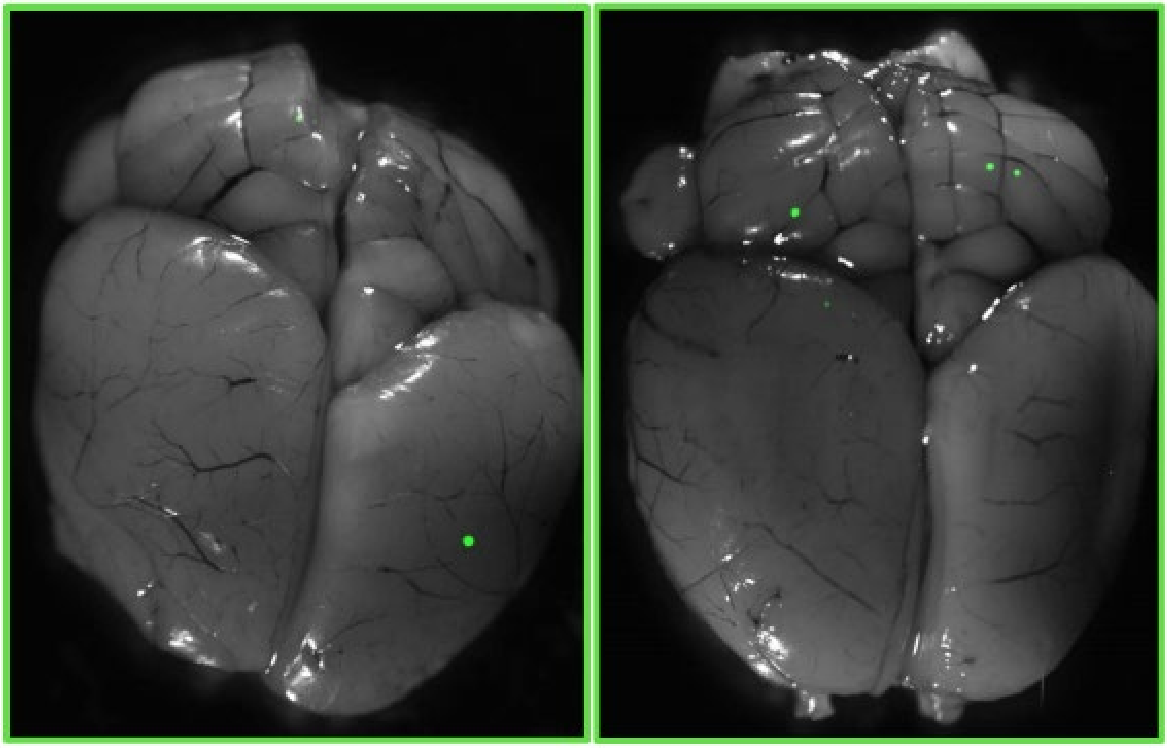

Supplementary Figure S1. **Representative images of brain metastases at 4 weeks after tumor-cell injection**. SCID/Beige mice were injected via tail vein with 5 × 10^5 MDA-IBC3 cells labeled with green fluorescent protein (GFP); 4 weeks later, the mice were killed and the brains excised and photographed with an AZ100 Nikon fluorescent stereomicroscope at 20X magnification. Metastases were visualized by merging a brightfield photograph of the organ with the GFP expression profile (adjusted for background autoflueorescence) with the Nikon NIS-Elements software.

Supplementary Table S1. Presence of Brain Metastases in Overall Study.

Radiation Dose	Time of Brain Irradiation	Incidence of Brain Metastases at 4 Weeks and 8 Weeks, No. (%)	*P* Value vs. 5-Days-After Condition
0 Gy	---	19/23 (83%)	0.0002
4 Gy	2 d before injection	20/20 (100%)	0.0001
4 Gy	5 days after injection	5/20 (25%)	---
4 Gy	3 weeks after injection	14/17 (83%)	0.0008
4 Gy	6 weeks after injection	7/8 (88%)	0.004

Supplementary Table S1. Presence of Brain Metastases in Overall Study. SCID/Beige mice) were injected via tail vein with 5×10^5 MDA-IBC3 cells and treated or not treated with whole-brain irradiation at the indicated times. At 4 weeks or 8 weeks after tumor-cell injection, the mice were killed and their brain excised and examined for the presence of metastases as described in Supplementary Fig. S1. Mice given whole-brain irradiation at 5 days after tumor-cell injection had significantly fewer brain metastases than each of the four other treatment groups. Brain metastases seemed to be more prevalent in the 2-days-before injection group than in the 3-weeks-after group, but this apparent difference was not statistically significant (P=0.09). Percentages between groups were compared with Fisher's exact test.

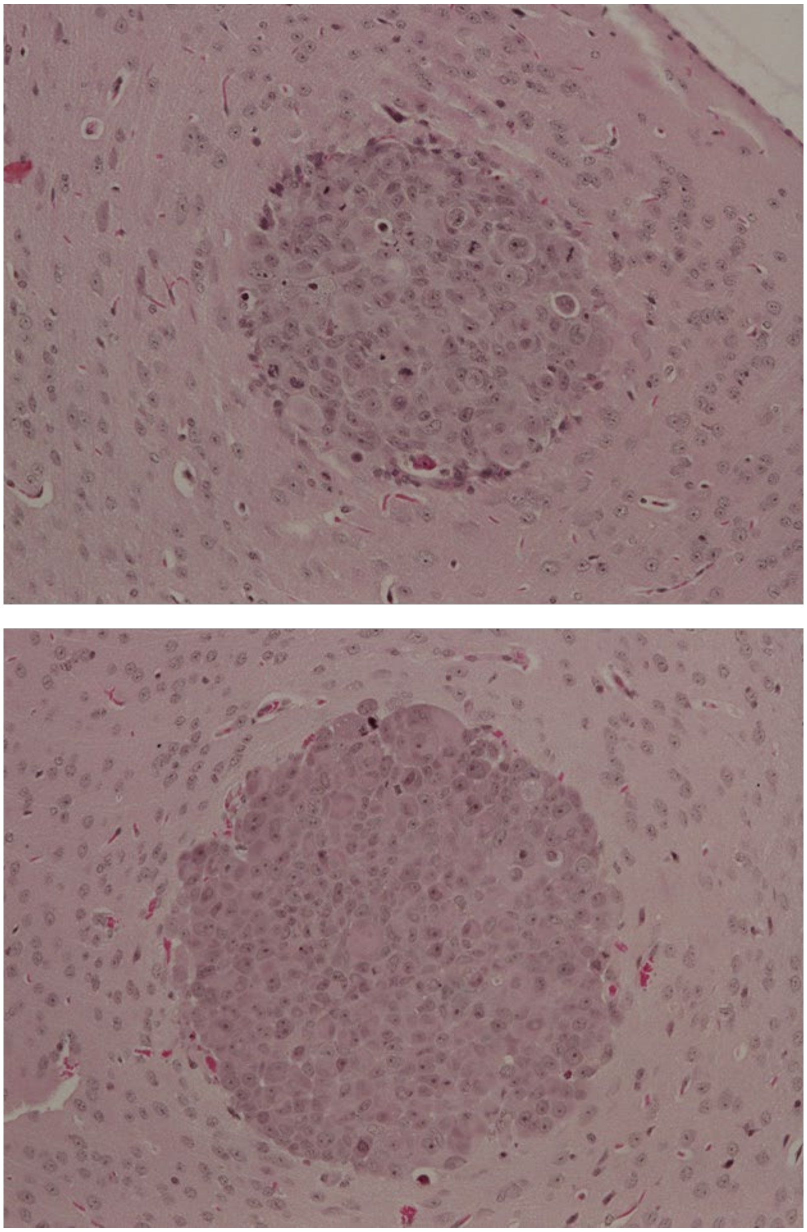

Supplementary Figure S2. Sections of brain tissue from mice sacrificed at 8 weeks after tumor-cell injection were formalin-fixed , paraffin-embedded, and stained with hematoxylin & eosin. Shown here are two representative images of brain metastases, viewed at 10X magnification.

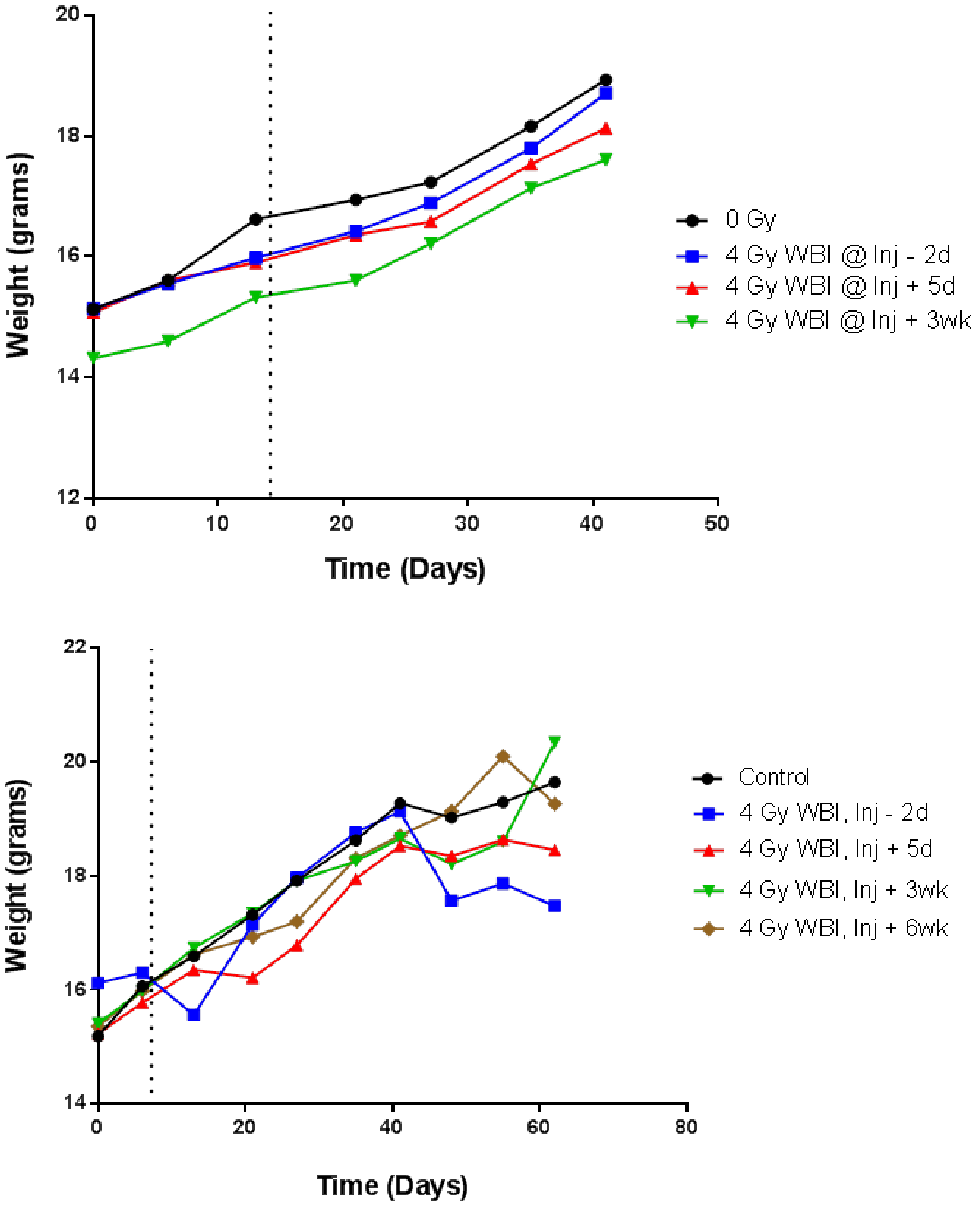

Supplementary Figure S3. Changes in mouse body weight over time. In each of the treatment groups at the 4-week endpoint (top panel), the weight of mice steadily increased over time. However, by 8 weeks (bottom panel), the mice had steadily gained weight until about 5 weeks after cell injection, after which the mean weights began to either decline or plateau. However, no correlation was found between the magnitude of weight loss and the brain metastatic tumor burden in individual mice (data not shown). The dotted lines represent time of tail-vein injection of MDA-IBC3 cells. Error bars not shown.
